# The Chemical Basis of Pharmacology: An Introduction to Pharmacodynamics Based on the Study of the Carbon Compounds

**Published:** 1908-03

**Authors:** 


**The Chemical Basis of Pharmacology: an Introduction to Pharmacodynamics based on the Study of the Carbon Compounds.** By Francis Francis, D.Sc., Ph.D., and J. M. Fortescue-Brickdale, M.A., M.D. (Oxon.). Pp. xii., 372. London: Edward Arnold. 1908.

As in the decorative arts there have been long periods of remarkable progress due to the separate study of individual branches, such progress leaving the student time to study only the barest rudiments of the other branches whilst he confines his practice to one alone, so in medicine the growth of specialism has been the means of enormously advancing our knowledge. But it becomes necessary that various branches should be again linked together, in order that full advantage may be taken of the increase due to separate growth, and each branch may profit by the separate growth of the others; it is the need of "linking up" that has called this book into existence, and by the co-operation of an expert in chemistry and an expert in pharmacology the ideal method has been followed, and the authors are to be heartily congratulated upon the result.

Let us say at once that the task before the authors has been one of exceptional difficulty. It is true, and duly acknowledged by the authors, that the ground has been to some extent prepared for them by Fraenkel's *Arzneimittelsynthese* and Brunton's *The Relation of Chemical Structure to Physiological Action*, but the present authors have boldly adopted, as far as is at present usefully possible, an arrangement that follows the usual lines of text-books on organic chemistry, with the result that we have now a systematic and clear exposition of the principal facts of our present knowledge of the subject in the handy compass of a book of less than four hundred pages.

The literature on the pharmacological properties of the numerous modern synthetic drugs is enormous, widely spread through the journals of all countries, and not easy to estimate at its true value, but these difficulties have been ably attacked and most creditably surmounted. As to the difficulty in estimating the value of the various drugs, there is throughout the book a wholesome restraint from forcing the therapeutical or physiological action of drugs to fit the theoretical requirements of their chemical formulæ, and, as the title of the work suggests, the principal stress is laid upon the physiological action of a drug rather than upon its therapeutical action. Whilst, as the authors themselves state, "clinical experience is the only test of the value of a drug," there must have been great temptation to consider a drug useful or useless according to its chemical structure, a temptation very successfully rejected on the whole. We note, however, a small slip in this direction, where they state that the liquid nonvolatile hydro-carbons "are without physiological action, and pass through the body unaltered. Hence the uselessness of petroleum emulsion . . . as a drug." One might as well say that because oil will not chemically unite with water, nor dissolve in it, and has been proved to have no obvious action on calm water, it is therefore utterly useless to pour it upon troubled waters. But on the whole the numerous pitfalls which await those who approach the subject from a purely chemical and physiological standpoint have been avoided in a manner worthy of very high praise. Thus the authors have avoided digressions into the "*Seitekette*" theory, although this must have had great attractions for them, or the varying action of certain drugs when given in large or small doses. They have by this restraint been able to confine themselves to a clear statement of the ascertained facts on the relationship of chemical structure to physiological action. As to the arrangement adopted, the chemistry of each group of substances is shortly described, and then the physiological characters are dealt with, and the relationship shown. Although the first glance at the book, from the profusion of formulæ, might possibly discourage those whose knowledge of organic chemistry has atrophied from disuse, this very profusion, in conjunction with the lucid treatment of the chemistry, is of the greatest assistance, and few will find themselves unable to follow the authors through these 360 pages. Indeed, the clearness and ease of expression is a striking and very welcome feature of this book.

The book does not pretend to be exhaustive, for their object would not have been attained so successfully had the underlying facts been smothered by a too great wealth of detail. Thus we find mention of the properties of allyl-thio-urea, or Thiosinamine, but none of the combination with sodium salicylate known as Fibrolysin, the beneficial action of the latter depending entirely upon the Thiosinamine portion. The majority of recent synthetic drugs of any importance will be found here; thus the practitioner will find Bromural and Veronal, and is hardly likely to require a reference to Broponal, although he may find its formula.

One great value of the systematic treatment of any subject is that it shows up clearly the important gaps in our knowledge, and suggests research in those directions, and here we have numerous groups thrown up in this relief, such as the group of the glucosides, where there is a large field for research in bio-physical and bio-chemical directions.

There is no doubt that the influence of this book will be widely felt. It gives a definite object and aim to the medical student in his study of organic chemistry, and definitely links up that subject in his mind to physiology, giving it a higher value in his curriculum than he or his teachers are inclined to give it at present; it will greatly influence the teaching of pharmacology and therapeutics; it will stimulate research, and be of inestimable assistance to the manufacturers of synthetic drugs in this country; and by its aid the practitioner may form some judgment upon the probable properties of these new drugs.

Professor Francis Francis and Dr. J. M. Fortescue-Brickdale have written a very valuable book, and the Bristol Medical School may well be proud of it, for it is the standard book in the English language upon the subject with which it deals.

